# Editorial: RNA-chromatin interactions: Biology, mechanism, disease and therapeutics

**DOI:** 10.3389/fgene.2022.1069427

**Published:** 2023-01-12

**Authors:** Prabhu Mathiyalagan, Luciano G. Martelotto, Samir Ounzain, Assam El-Osta, Shizuka Uchida

**Affiliations:** ^1^ Center for Human Genetics and Genomics, New York University, New York City, NY, United States; ^2^ Adelaide Centre for Epigenetics, South Australian Immunogenomics Cancer Institute, The University of Adelaide, Adelaide, SA, Australia; ^3^ HAYA Therapeutics, Lausanne, Switzerland; ^4^ Department of Diabetes, Central Clinical School, Monash University, Melbourne, VIC, Australia; ^5^ Center for RNA Medicine, Department of Clinical Medicine, Aalborg University, Copenhagen, Denmark

**Keywords:** ncRNA (non-coding RNA), RNA, chromatin, transcription, epitranscriptome

## Introduction

From the development to disease, long non-coding RNAs (lncRNAs) regulate chromatin landscape ultimately influencing gene transcription, X-chromosome inactivation, RNA-mediated epigenetic inheritance, DNA methylation and genome stability through maintenance of active and silent chromatin state. LncRNAs function through establishing and maintaining stable RNA-chromatin interactions at a specific genomic locus through RNA-protein and/or RNA-DNA interactions. Growing evidence underscores the significance of lncRNA sequence, structure and epitranscriptomic modifications as potential mediators of RNA-chromatin interactions. Whether lncRNA sequence, structure and epitranscriptomic marks, either alone or in a particular combination, act as specific molecular code governing RNA-chromatin interactions needs to be elucidated. The future of lncRNA research and its therapeutic success relies on mapping the interplay among lncRNA sequence, structure and epitranscriptome. Further, it is important to understand how these features, alone or in a particular combination, stabilize RNA-chromatin interactions ultimately influencing gene expression and function.

## Non-coding RNAs space it up

As we enter the space age, it is important to understand how the non-gravitational environment affects our health (Demontis et al., 2017) In this regard, Bisserier et al. provide novel insights into potential roles of exosomal lncRNAs in determining the effects of radiation after spaceflight. As exosomal RNAs are known to mediate cellular communication, Bisserier et al. provide new experimental data on exosomal RNAs obtained from peripheral blood plasma of three astronauts who flew on various shuttle missions between 1998 and 2001. In their transcriptome analysis of exosomal RNAs during spaceflight, they report several differentially expressed lncRNAs in exosomes isolated from astronauts at different timepoints during their spaceflight. These results also provide new insights into the diagnostic and prognostic value of astronauts-derived exosomal lncRNAs as emerging ncRNA biomarkers and highlight the potential role for exosomal lncRNAs in health risks associated with spaceflight.

## Non-coding RNAs at the center of liquid-liquid phase separation

For a biochemical reaction to occur in a cell, the interacting partners must be present in the close proximity (Stanton et al., 2018). To address this point, Somasundaram et al. provides an in-depth analysis of lncRNAs with specific focus on their role in establishing and maintaining biomolecular phase-separation. Formation of membrane-free ribonucleoprotein condensates resulting in intracellular compartments by liquid–liquid phase separation or condensation is a critical phenomenon that compartmentalizes lncRNA and other macromolecules separate from others. In this *Review* article, Somasundaram et al. further provides a deeper understanding of phase separation, physio-chemical properties of such biomolecular condensates, their composition and the role of RNAs in driving the formation and stabilization of biomolecular condensates. Particularly, the *Review* highlights several lncRNAs, which are known to act as “the anchor” using their specific intrinsic sequence motifs within the condensates. Furthermore, the authors provide some recent evidence for lncRNAs working through phase separation in association with diseases, such as cancer, further highlighting the significance of condensate biology in cellular physiology and argue why better understanding of condensates would enhance therapy. Taken together, this review article is an excellent addition and an insightful summary of new developments in the field of ncRNA and phase separation.

## Epitranscriptome connects the non-coding and coding transcriptomes

To date, over 170 RNA modifications are known across organisms (Boccaletto et al., 2018), which opened up a new field of study called epitranscriptomics. As epitranscriptomic marks are found in all types of RNA, there is a growing interest to study how epitranscriptomic marks influence the functions of lncRNAs. To introduce readers to this new existing field of study from the perspective of lncRNAs, Lee O. Vaasjo summarizes chemical changes to RNA as a critical regulator of mRNA regulation. RNA modifications, particularly in the form of N6-methyladenosine (m^6^A) methylation, have been shown to be integral for RNA metabolisms of mRNAs and for several lncRNAs. For mRNAs, m^6^A modifications are specifically regulated through distinct mechanisms occurring within 3′-UTR or coding regions. However, little is known on how m^6^A modifications are regulated within 5′-UTRs of mRNAs. In this Research Topic, Vaasjo summarizes elegantly how m^6^A methylation regulates 5′-UTRs of mRNA through lncRNAs. Specifically, the author provides recent examples on how several lncRNAs can target specific sites within 5′-UTRs of mRNAs to regulate m^6^A patterning within 5′-UTRs of mRNAs. Furthermore, the author highlights a critical epigenetic crosstalk mediated by lncRNAs through direct interactions of lncRNAs with chromatin remodeling proteins in determining m^6^A pattern within 5′-UTRs of mRNAs. This *Review* highlights an important crosstalk occurring between lncRNAs and the m^6^A modifying machinery in the nucleus; addressing one of the key mechanisms by which lncRNAs not only working at the level of gene expression but also at the epitranscriptomics level thereby regulating the expression of coding transcriptome. This *Review* can be regarded as an excellent summary of novel findings that underscores an important interplay between the non-coding (ncRNA) transcriptome and the coding (mRNA) transcriptome through complex regulation of epitranscriptomic mechanisms.

## RNA-modifying enzymes and RNA-binding proteins at the heart of mitochondria

Mitochondria are dynamic powerhouses of the cell that are important for many cellular activities (Tilokani et al., 2018). As the heart must work restlessly to sustain the life of an organism, the proper functionality of mitochondria is essential as dysregulation of mitochondrial functions lead to heart failure. Ziemann et al. tackles the problem of identifying ncRNAs and RNA-binding proteins (RBPs) that may be associated with hearts of dilated cardiomyopathy (DCM). Ziemann et al. report significant changes in expression of several genes encoding ncRNAs, such as ribosomal RNAs (rRNA), and further classify that downregulation of several RBPs as a specific feature of hearts in DCM. Although roles for majority of ncRNAs remain unexplored in mature adult tissues, such as in the heart, Ziemann et al. elegantly show that dysregulated expressions of several RBPs are present in hearts of DCM. In particular, the changes were observed in expression for gene sets of mitochondrial (mt)-rRNA processing, aminoacyl-tRNA synthases, and mitoribosome subunits for in-situ protein synthesis. The authors identified several enzymes such as mitochondrial rRNA methyltransferases (e.g., Mrm1-3, Nsun4 and Trmt10c) that regulate rRNA epitranscriptome and mitochondrial ribonucleases (e.g., Prorp, Elac2) as differentially regulated genes in DCM. Whether these changes in expression to rRNA methyltransferases and RNases have transcriptome-wide impact on coding and non-coding RNAs needs further investigation. Interestingly, Ziemann et al. report downregulation of several mtRBPs, including Slirp, Gadd45GIP1, Dap3, Ptcd1-3, Lrpprc, Grsf1 etc, which are involved in rRNA modification and formation of mitoribosome. However, how these RBPs influence the pathophysiology of DCM remains unknown. Together, these new results from Ziemann et al. provide important new evidence indicating changes in expression of enzymes and proteins potentially involved in ribosomal epitranscriptome in DCM hearts.

## Conclusion

In summary, this Research Topic provides an important overview on RNA-based molecular mechanisms that are not only of fundamental importance but also of therapeutics. Research articles as well as Reviews published in this Research Topic cover a broad range of mechanisms involving RNA-chromatin interactions. These mechanisms provide important insights to uncover ncRNA function and a deeper understanding of RNA-chromatin interactions will undoubtedly begin a new era in RNA-based therapeutics.
